# Potential of *Marava arachidis*, a Newly Recorded Earwig Species in Egypt as a Biological Control Agent of *Rhipicephalus annulatus* Tick in Laboratory

**DOI:** 10.3390/insects13100934

**Published:** 2022-10-15

**Authors:** Shawky M. Aboelhadid, Abdel-Azeem S. Abdel-Baki, Sahar M. Gadelhaq, Walid H. Hassan, Lamjed Mansour, Saleh Al-Quraishy, Yoshitaka Kamimura, Chow-Yang Lee, Asmaa A. Kamel

**Affiliations:** 1 Parasitology Department, Faculty of Veterinary Medicine, Beni-Suef University, Beni-Suef 62511, Egypt; 2 Zoology Department, Faculty of Science, Beni-Suef University, Beni-Suef 62521, Egypt; 3 Parasitology Department, Faculty of Veterinary Medicine, Minia University, Minia 61519, Egypt; 4 Departments of Bacteriology, Mycology and Immunology, Faculty of Veterinary Medicine, Beni-Suef University, Beni-Suef 62511, Egypt; 5 Zoology Department, College of Science, King Saud University, Riyadh 11451, Saudi Arabia; 6 Department of Biology, Keio University, 4-1-1 Hiyoshi, Yokohama 223-8521, Japan; 7 Department of Entomology, University of California, Riverside, CA 92521, USA

**Keywords:** earwig, *Marava arachidis*, identification, predation, *Rhipicephalus annulatus*

## Abstract

**Simple Summary:**

Based on both the morphological and molecular data of the earwig samples collected from a bakery in Beni-Suef, Egypt, we identified the species as *Marava arachidis* (Spongiphoridae). The predation capability of *M. arachidis* against the eggs and larvae (in-ground stages) of the *Rhipicephalus* *annulatus* tick under laboratory conditions was assessed. Laboratory findings revealed that this species has predation capability on *R. annulatus* tick eggs and larvae.

**Abstract:**

In Egypt, only five species of Dermaptera (earwigs) have been reported. Based on both the morphological and molecular data of the earwig samples collected from a bakery in Beni-Suef, Egypt, we identified the species as *Marava arachidis* (Spongiphoridae), a cosmopolitan species with no prior records in Egypt. The current study was designed to analyze its predation capability on newly emerged eggs and larvae of the *Rhipicephalus annulatus* tick. A laboratory functional response study was set up by applying a predation test with various predator-prey ratios as treatments. This experiment was applied using the undefined mix of collected earwigs and the laboratory-collected eggs and the larvae of *R. annulatus*. The laboratory results showed that the mean number of predated tick eggs was 18.64 ± 11.18 in 24 h under the highest predator-prey ratio (1:10) examined, accompanied by 12.04 ± 4.38 broken but unconsumed eggs. Moreover, *M. arachidis* predated an average of 12.32 ± 9.07 tick larvae per day. In contrast, the mean dead larvae increased to 38.4 ± 2.30 per day with the highest predator density (1:10). The number of eggs and larvae consumed increased with the predator density. A linear relationship was detected between earwig density and the consumption rates of tick eggs (R^2^ = 0.99; *p* = 0.0001) and larvae (R^2^ = 0.96; *p* = 0.003). In conclusion, *M. arachidis* was first recorded in Egypt. This earwig has predation capability on *R. annulatus* tick eggs and larvae.

## 1. Introduction

Earwigs (Insecta: Dermaptera) are a polyneopteran insect order with approximately 2000 species [1,2,3,4]. All the known earwig species are incorporated into 12 families [5]. Of these, only four families were previously recorded in Egypt. These families are Anisolabididae, Labiduridae, Spongiphoridae (=Labiidae), and Forficulidae [6]. Although the bone-house earwig, *“Marava arachidis*,” is a widely distributed species [7], it had previously not been recorded in Egypt. Little is known about the origins of this species [8]. It was first discovered in California in 1920 and has been documented in the state ever since [9]. This species is known to be ovoviviparous: females keep well-developed embryos encased in thin but complete egg envelopes until a few minutes before hatching [8,10,11]. Herein, *M. arachidis* was recorded in the bran in a local bakery. The present study’s first aim was to confirm this species’ identification for the first time in Egypt.

Chemical acaricides are the most common and effective method of tick control at present. However, this approach has various drawbacks, particularly environmental and public health adverse effects and the development of resistant tick strains [12] (Samish and Rehacek 1991). Therefore, it is necessary to search for alternative and sustainable control strategies, including the potential use of natural enemies such as predators, pathogens, or parasitoids for the biological control of ticks [12] (Samish and Rehacek 1991). There are three types of tick predators; ants, beetles, and other arthropods [13]. Arthropod predators are often nonspecific feeders, and the host density of both predator and prey in an ecological niche has a major potential impact on the utility of arthropod predators as a biocontrol agent [14]. Following this perspective, Goulart et al. [15] studied the predation of earwigs (*Forificula* sp.) on *Boophilus microplus* eggs. Moreover, Verissimo [16] reported that a single earwig representative preyed on approximately 1800 *B. microplus* eggs in 10–12 days. *Marava arachidis* is an earwig with worldwide distribution, mainly in tropical regions [17]. This earwig is commonly present in stored animal rations and many animal vivariums [8]. In addition, Abramson et al. [18] found that *M. arachidis* can feed on aphids, use the fennel plant as shelter, and climb the fennel plants to feed on fennel nectar. In the present study, *M. arachidis* was recorded in the bran of a local bakery. The diversity of habitats in which *M. arachidis* was recorded reflects the importance and potential of this insect as a predator in programs of integrated pest management. In addition, *M. arachidis* offers many benefits as a biological control, including the ease of laboratory rearing, the availability of artificial diets, and the ability to rear large numbers of specimens suitable for field use [18]. The second aim of the present study was to investigate the predation capability of *M. arachidis* against eggs and larvae (in-ground stages) of the *Rhipicephalus annulatus* tick under laboratory conditions.

## 2. Material and Methods

### 2.1. Source of Study Earwigs

The earwig insects were obtained from a local bakery in the spring of 2019 in Beni-Suef, Egypt. The owner received a complaint of bran infestation by insects of unknown origin. The owner collected about half a kilogram of infested bran and brought it to the Parasitology Department, Faculty of Veterinary Medicine, Beni-Suef University. The insects were isolated by bran sieving. The isolated insects were then maintained in the lab for subsequent use.

### 2.2. Morphological Identification

The morphological identification of the present earwig was carried out using an SZX16 stereo-microscope (Olympus, Tokyo, Japan). The “Microscope mode” and “Focus-stacking sub-mode” of a Tough-TG5 digital camera (Olympus) were also used to obtain composite images for the external morphological criteria of the present earwig. After softening in hot water, the male genitalia were extracted from a dried male specimen under a stereo microscope. After mounting on a glass slide with phosphate-buffered saline (PBS), genital structures were observed and photographed under a BX53 differential interference contrast (DIC) microscope (100–400×; Olympus) equipped with a Pen e-pl1s digital camera (Olympus). Based on photographs taken under a DIC microscope, the digital composition of the selected parts of each image in focus was performed using Combine ZP Image Stacking Software [19].

### 2.3. Molecular Identification

DNA was extracted from a single leg of a fixed specimen in 70% ethanol using QIAamp DNA Mini Kit (Qiagen, Germany) according to the manufacturer’s instructions. The extracted DNA was processed for the amplification of the partial sequence of cytochrome oxidase I (COI) [20] using the primers LepF (5′-ATTCAACCAATCATAAAGATATTGG-3′), and LepR (5′-TAAACTTCTGGATGTCCAAAAAATCA-3′) amplified the target 658-bp fragment of COI [21].

The PCR was carried out in 20 µL of final reactions volume in a T100^TM^ Thermocycler (Bio-Rad, Singapore) and contained 1 µL of DNA, 0.5 µL of each primer (10 pmol), and 10 µL of GoTaq^®^ Master mixes 2X (Promega, New Delhi, India). The amplification conditions were as follows: initial denaturation step at 95 °C for 3 min, and then subjected to 35 cycles of 30 s at 94 °C, 30 s at 50 °C, and 60 s at 72 °C, and a final extension for 10 mn at 72 °C. After amplification, the PCR products were purified using EXO-SAP (New England Biolabs) and sequenced using the BigDye Terminator v.3.1 Sequencing Kit (Applied Biosystems, Foster City, CA, USA) in an ABI Prism 3730 XL Genetic Analyzer (Applied Biosystems).

For comparison with the earwig samples from Egypt, total genomic DNA was extracted from an ethanol-preserved male sample of a Malaysian population of *M. arachidis* studied by [11,22]. Total genomic DNA was extracted from three legs of an ethanol-preserved male sample using a DNeasy Blood and Tissue Kit (Qiagen, Hilden, Germany) according to the manufacturer’s instructions. The PCR amplification of a mitochondrial cytochrome oxidase subunit I (COI) region, which is widely used for DNA barcoding in many invertebrates [23], was performed using a T100TM thermal cycler (Bio-Rad Laboratories, Inc., Hercules, CA, USA) and a primer set (LCO1490 and HCO2198: [23]. The PCR reactions were conducted in a 20 μL volume containing the primers (1 μL of 10 μM each), 10 μL 2× PCR buffer, 4 μL dNTPs (2 mM each), 0.4 μL KOD FX Neo DNA polymerase (1.0 unit/μL; Toyobo, Osaka, Japan), and 1 μL of genomic DNA. The PCR temperature profile consisted of 2 min at 94 °C, then 35 cycles of 15 s at 94 °C, 15 s at 51 °C, and 15 s at 72 °C, followed by a final extension for 6 min at 72 °C. Sequencing was provided as a custom service by Eurofins Genomics, Inc. (Tokyo, Japan). The chromatograms were visually checked and manually edited where appropriate. After eliminating the primer sequences, the COI sequences were deposited in GenBank.

Multiple sequence alignments were conducted with ClustalW [24], implemented in MEGA11 software [25] using the default parameter settings. The calculation of the *p*-distance between the Egyptian and Malaysian samples was performed by MEGA11.

### 2.4. Potential Predation of Earwig on R. annulatus Tick Stages

#### 2.4.1. *R. annulatus* Eggs and Larvae Preparation

Fully engorged adult females of *R. annulatus* were collected from naturally infested cattle in different localities in the Beni-Suef province, Middle Egypt (29°03′60.00″ N 31°04′60.00″ E). The ticks were transported to the Parasitology Lab at the Faculty of Veterinary Medicine, Beni-Suef University. The ticks were identified according to [26]. Female ticks were incubated at 27 °C and ≥80% relative humidity in a BOD incubator for egg deposition. Subsequently, the deposited eggs were set at 27 °C and ≥80% relative humidity for hatching. The hatched larvae were used in the predation test.

#### 2.4.2. Predation Test

The predation effects of *M. arachidis* on the eggs and larvae of *R. annulatus* were studied under laboratory conditions at the Parasitology Department, Beni-Suef University, Egypt, in a BOD incubator at 20 ± 1 C with 60 ± 10% relative humidity [27]. To evaluate the predation rate, five predator-prey ratios were studied. The number of eggs and larvae was kept constant at 100 per Petri dish, while the predator number was varied. The numbers of earwigs per Petri dish were 1, 3, 5, 7, and 10, resulting in predator-prey ratios of 1:100, 1:33, 1:20, 1:14, and 1:10, respectively. Each ratio was replicated five times [28].

For the predation experiment, the earwigs collected from the bakery were first acclimatized for 24 h, then starved for 24 h, and finally allowed to predate for 24 h. In the acclimatization stage, the earwigs were placed with tick eggs or larvae for 24 h to adapt them to this type of feeding. Then, the earwigs were left to starve for 24 h to standardize the predation conditions [28].

**Figure 1:**
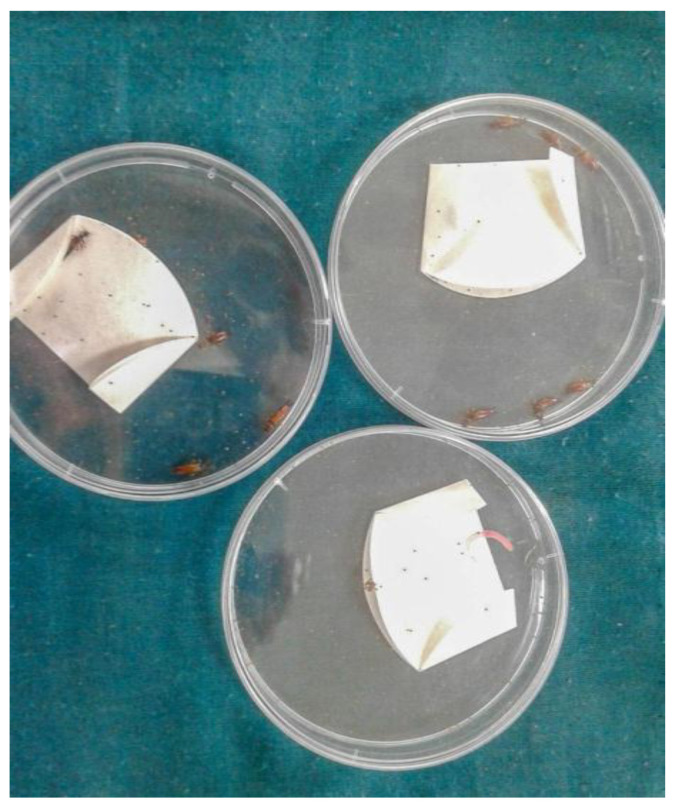
Petri dishes containing packet filter paper as the setup for eggs and larvae predation.

Afterward, an undefined mix of adult earwigs was transferred to Petri dishes containing 100 tick eggs and other Petri dishes containing 100 tick larvae in a filter paper packet (each experiment was performed separately) (Figure 1). After 24 h, the numbers of eggs and larvae were counted under a stereo microscope to evaluate the predation rate. The broken and eaten eggs were considered predated by the earwigs: predated eggs = total egg number (100)–intact eggs. Meanwhile, the predated larvae were evaluated: predated larvae = 100 − (live larvae + dead) [27].

### 2.5. Statistics

Using linear regression, the predatory activity against tick eggs and larvae was evaluated by studying the relationship between the number of eggs and larvae consumed (or broken) and the predator density. ANOVA and Duncan’s multiple range tests were applied to determine differences between means (α = 0.05).

## 3. Results

### 3.1. Identification of the Present Earwig

**Figure 2:**
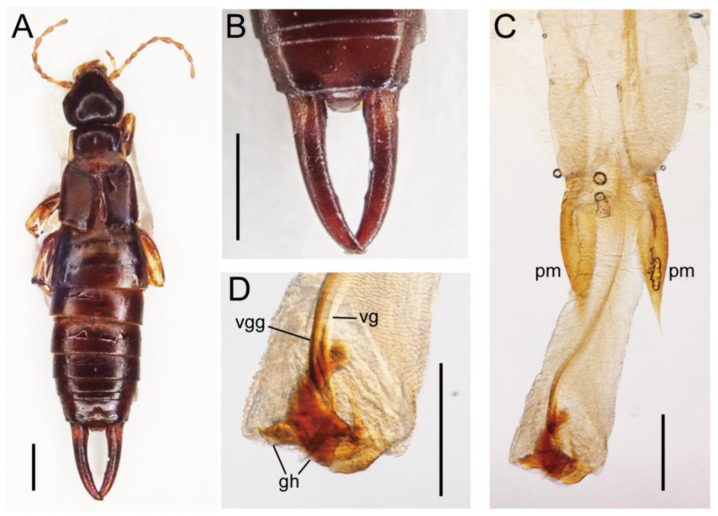
Habitus (**A**), pygidium and forceps (**B**), genitalia (**C**: distal part), and penis lobe (**D**) of male *M. arachidis*, collected from a bakery in Beni-Suef city, Egypt. Abbreviations: gh, genital hook; pm, paramere; vg, virga; vgg, virgal guide. Scale bars: 1 mm in (**A**,**B**); 250 µm in (**C**,**D**).

The body of the earwig samples examined in the present study was not flattened dorso-ventrally. The antennal segments were conical or subpyriform, and the eyes were longer than the first antennal segment but shorter than the distance between the eye and mouth parts (malar space). The hind wings are wanting, but the tegmina were well developed without a longitudinal keel at the lateral margins (Figure 2A). The second tarsal segment was normal and not elongated under the third segment. There were no conspicuous tarsal arolia between the claws. The ultimate abdominal tergite was almost rectangular, being wider than long. The male forceps were simple and not undulated. The pygidium showed two small tubercules on each side (Figure 2B).

In the male genitalia, only a single penis lobe was present, which encloses an elongated virga associated with a guiding structure and a characteristic hook composed of a pair of unequally-shaped sclerites (Figure 2C,D). The parameres were acuminate and not undulated (Figure 2C).

The DNA barcoding region of the Egyptian sample showed only a 1.55% sequence difference (10 out of 645 bases compared) from *M. arachidis* collected in Malaysia. The accession number for the Malaysian *M. arachidis* is “LC15968”.

### 3.2. Predation Activity of Earwigs against Ticks Eggs and Larvae

**Figure 3:**
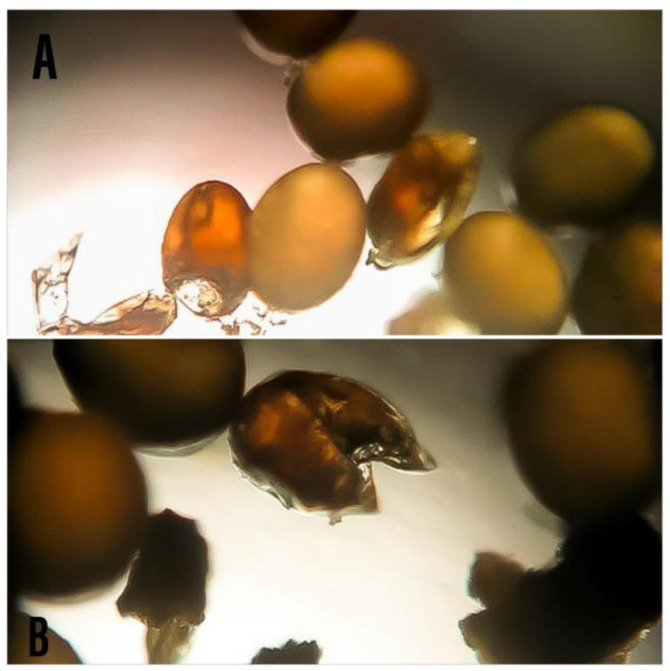
Broken eggs (**A**,**B**) as evident of predation of earwigs on *R. annulatus* eggs.

**Figure 4:**
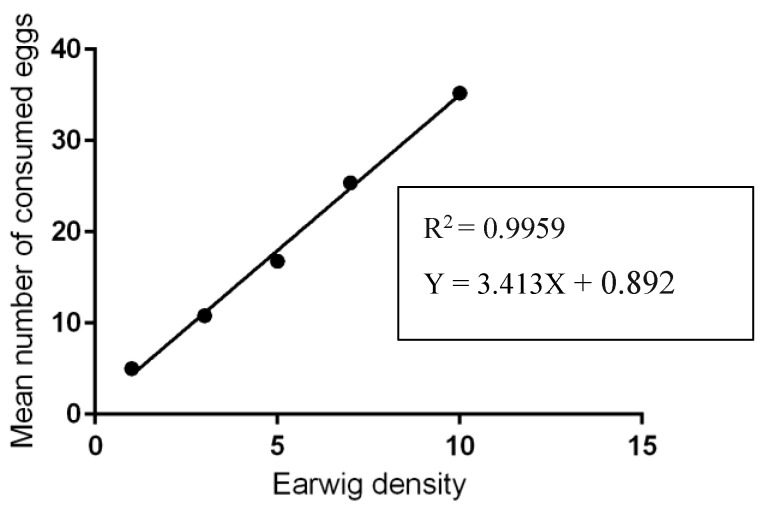
The number of eggs consumed by adult earwigs present in each ratio after 24 h. The plain line is the estimated linear regression (R^2^ = 0.99, *p* = 0.0001).

**Figure 5:**
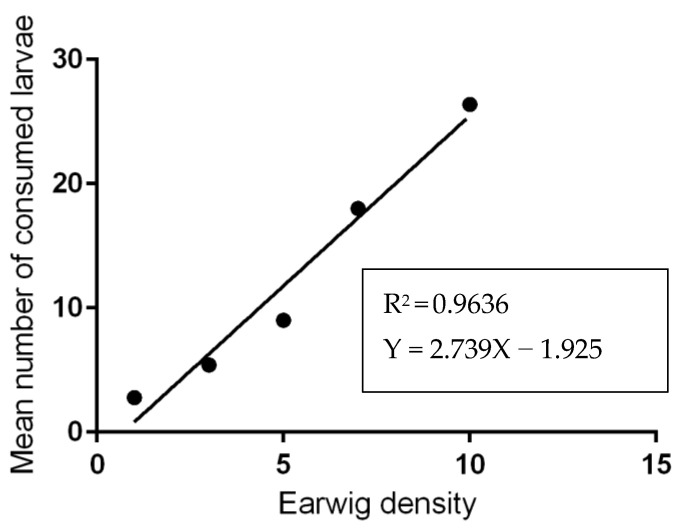
The number of larvae consumed by adult earwigs present in each ratio after 24 h. The plain line is the estimated linear regression (R^2^ = 0.96, *p* = 0.003).

The laboratory results showed that an undefined mix of adult earwigs was fed on 5.00 ± 1.41 to 35.2 ± 3.96 tick eggs; meanwhile, the number of broken eggs ranged from 6.4 ± 1.51 to 17.2 ± 4.60 eggs (Table 1, Figure 3) after 24 h, depending on the predator-prey ratio. For tick larvae, adult earwigs predated about 2.8 ± 1.48 to 26.4 ± 2.70 larvae after 24 h, while the number of dead larvae reached 38.4 ± 2.30 larvae after 24 h, depending on the predator-prey ratio (Table 2). The number of predated eggs and larvae depended on the predator-prey ratio. The effect of predator density on the number of prey consumed was significant (*p* < 0.05). The highest number of consumed eggs or larvae was detected at the predator-prey ratio of 1:10, and the lowest was encountered at the 1: 100 ratio. In general, the total number of consumed eggs or larvae after 24 h increased as the density of predators increased (Table 1 and Table 2). Linear relationships were observed between the earwig densities and the consumption rates of the ticks’ eggs (R^2^ = 0.99, *p* = 0.0001: Figure 4) or larvae (R^2^ = 0.96; *p* = 0.003: Figure 5).

## 4. Discussion

The presence of a single penis in the male genitalia and normal second tarsal segments without elongation under the third tarsal segments supported that the present earwig species belongs to Spongiphoridae (=Labiidae) [1,7]. The present earwig samples also showed lateral margins of the tegmina without a longitudinal keel, claws without conspicuous tarsal arolia, a body not flattened dorso-ventrally, eyes longer than the first antennal segment but shorter than the distance between the eye and mouth parts (malar space), ultimate abdominal tergite of a rectangular shape, conical or subpyriform antennal segments, and parameres and forceps being simple and not undulated, all of which reinforced its recognition as a member of the genus *Marava* Burr (Spongiphorinae) [11].

The genus *Marava* is common, especially in the Neotropical region, and ca. 45 species have been reported around the world [4,7]. However, the characteristic shape of the pygidium with two small tubercles on each side, the acuminate parameres, and the elongate virga accompanied by a guiding structure and a distinctive hook composed of a pair of uniquely shaped sclerites indicate that the present earwig is *M. arachidis* (Yersin) [7]. Analysis of the DNA barcoding region also supported this view as the Beni-Suef, Egypt sample showed only a 1.55% sequence difference (10 out of 645 bases compared) from the *M. arachidis* collected in Malaysia [11,22].

Little is known of the Dermapterous fauna of Egypt. Only five species belonging to four genera of four families (Anisolabididae, Labiduridae, Spongiphoridae, and Forficulidae) have been reported to represent the Dermapterous fauna of Egypt [6]: *Euborellia annulipes* (Lucas) (as *Anisolabis annulipes*), *Labidura riparia* (Pallas), *Labia minor* (L.), *Forficula auricularia* L., and *Forficula lucasi* Dohrn. *Marava arachidis* is a small species belonging to the family Spongiphoridae [11]. It is an Indo-Australian species that is well established in different parts of the world (Africa, Australia, Southern Asia, etc.) but occurs only sporadically in Britain and northern Europe, usually in water houses of imported organic materials [4,7]. This species might be imported to Egypt with goods transported by land, ship, or air [29]. Based on the authors’ knowledge and identification, this is the first report denoting the existence of the earwig *M. arachidis* in Egypt.

Acaricides have mostly been the effective means for controlling tick populations until recently. However, this approach has many drawbacks, particularly environmental contamination and the rising prevalence of acaricide resistance in many tick species [30]. Therefore, it is crucial to implement biological controls to lessen the need for pesticides. Earwigs are organisms with high predatory capacity and act as essential predators for several agricultural pests, particularly aphids and mites in the agricultural landscape [31]; therefore, earwigs can serve as effective biological pest control agents for several insect species [32,33]. In the present study, the earwig *M. arachidis* was reported for the first time in Egypt in bran in a local bakery. This species has been also recorded in other types of habitats; stored animal rations, many animal vivariums, and several agroecosystems [8,18].

Similarly, the habitat is suitable for the development of the non-parasitic phase of the *R. microplus* tick life cycle (eggs and larvae) [34]. Briefly, fully engorged female ticks dropped from the animals to the ground, and after that, ticks began to deposit eggs for 2 weeks. The deposited eggs hatched after 10–14 days, and the newly hatched larvae searched for the host to climb onto the animal to complete their life cycle. So, it is noted that eggs and larvae can stay on the ground for several days. This period is sufficient for them to come into contact with and be predated by the predators like earwigs. Furthermore, the utility of *M. arachidis* as a predator for tick control is reinforced by the ease of laboratory rearing, the availability of artificial meals, and the capacity for large-scale rearing. Based on the characteristics mentioned above, this study was designed to assess, under laboratory conditions, the predatory behavior of earwig *M. arachidis* on the eggs and larvae of *R. annulatus* tick.

The results showed that *M. arachidis* is a predator of the eggs and larvae of the *R. annulatus* tick. This is the first reported study of an earwig feeding on the larval stages of *R. annulatus* ticks. At least in the laboratory, the data showed that *M. arachidis* could be an efficient enemy of tick eggs and larvae either by eating them or attacking their bodies without eating, leaving them dead. Under the laboratory conditions, the adult earwig (*M. arachidis*) consumed about 35.2 ± 3.96 eggs with about 17.2 ± 4.60 broken ones per day at the highest predator-prey ratio of 1:10. In addition, *M. arachidis* predated an average of 26.4 ± 2.70 tick larvae with about 38.4 ± 2.30 dead ones per day under the highest predator-prey ratio examined (1:10). It was found that the number of consumed eggs and larvae increased as the density of predators increased. The use of earwigs as biological control agents has been investigated worldwide using various species [28,35,36,37,38,39]. According to Jiang and Kajimura [40], the predatory process by which earwigs attack their prey is carried out in the following steps: touching the prey’s body with its antenna, cutting it with its forceps, and consuming the contents with its mouth parts, then it looks for new fresh prey. However, not all of the processes must be completed. Earwigs, for example, totally consumed *Euwallacea interjectus* without cutting it with forceps. An unsuccessful predatory process was also observed: when earwigs touched *Xylosandrus brevis*, they cut its body, but the contents were not eaten. This could explain the presence of broken tick eggs and dead larvae in the current study that were not consumed.

The past literature presented a wide range of consumption rates by various earwig species, e.g., the earwig *Doru taeniatum* was proved to be an effective predator of red spider mite eggs [41], armyworms, aphids, and other soft-bodied insect pests in corn and sugarcane in the USA [42], and as an effective bio-control agent of faba bean pests in the USA [43]. Moreover, Buxton and Madge [31] found that an adult earwig and its third and fourth instars may eat 46–122 aphids (*Phorodon humili*) per day in Canada. What is more, Crumb and Bonn [44] revealed that an earwig could consume 2.5–5.2 *Acyrthosiphum spartii*, 3 *Aphis pomi*, and 5.2 *Eriosoma lanigerum* in the USA. The European earwig has been described as a significant predator of the diaspidid scale insect pest of kiwifruit in New Zealand [28]. It was found that after introducing earwigs in kiwifruit fields for eight weeks, they could feed on roughly 83 percent of the scale populations [45]. In addition, Logan et al. [45] reported that up to 40% of scale insects could be digested before harvest in autumn when earwigs are abundant in the kiwifruit canopy during the summer.

Despite the differences in prey species, our results were close to those of Kharboutli and Mack [46], as they detected that *F. auricularia* could consume 21.5 mites (Halotydeus destructor) and 18 aphids (*P. humili*) in Australia. In addition, Weiss and MacDonald [47] found that an adult earwig could feed on 8–9 *Aphis* sp. per day on wheat. The lower per-capita feeding rate observed in the present study, compared with the rates of other studies, could be attributed to the egg exoskeleton and the cuticle of larvae that provide protection against the earwig attack. Moreover, it was suggested that the differences in feeding rates recorded in various studies in Australia, the USA, Canada, and other locations were associated with the prey species [47]. Additionally, the differences in earwig sizes play an important role, as the larger the earwigs, the more prey consumed. That could be attributed to their higher activity of prey capture and the need for additional energy to maintain their comparatively larger mass [31]. Similarly, He et al. [48] found that heavier earwigs consumed significantly more apple leaf curling midge (ALCM) larvae than light ones in New Zealand. In many animal species, the relative body sizes of predator and prey play an essential role in their relationship [49,50].

Compared to the other female earwigs, which must care for eggs for several days to a few weeks, *M. arachidis* is an ovoviviparous species that deposit their eggs with developed embryos, and the first instar nymphs emerge from the eggshells 10–20 min after deposition [8,10,11]. Like viviparity, ovoviviparous species’ embryogenesis primarily occurs within the mother’s body. However, ovoviviparous embryos grow using materials reserved within the egg rather than consuming nutrients from the mother [51]. Due to its ovoviviparity, *M. arachidis* is likely more robust to disturbance during the egg-caring period, and thus the mass-rearing of them would be easier than oviparous species [22]. Therefore, their usefulness as a pest control agent may depend on the consumption rate and the ease of mass rearing.

Generally, the possibility of earwig predation on tick eggs and larvae is supported by previous observations. According to Hill et al. [52], some species of earwigs have a broad distribution across a range of agricultural environments. Moreover, earwigs can make multiple changes in their life-history traits in response to thermal regimes, climate, and human influence, as indicated by the case of the distribution limits of the European earwig, *Forficula auricularia*, which invaded Australia [52]. Additionally, *M. arachidis* gained experience in predation behavior, perhaps learning to search for alternative foods and how to hide. All these behaviors give earwigs a biological advantage in growing and maintaining their populations under different environmental conditions [53].

## 5. Conclusions

This is the first recorded mention of the earwig *M. arachidis* in Egypt. The present study provided the first basic information on the effectiveness of earwigs as a predator of *Rhipicephalus annulatus* ticks in Egypt (eggs and larvae). Although the earwig in the present study did not show remarkable levels of predation rates against ticks, it may aid in the natural control of tick infestations either by feeding on eggs and larvae or attacking their body with forceps. Therefore, further studies are needed to assess the potential of earwigs as predators of ticks.

## Figures and Tables

**Table 1:** The number of eggs consumed by adult earwigs present in each ratio after 24 h, under laboratory conditions.

Predator-Prey Ratios	Mean Number of Eggs Consumed by Earwigs	Mean Number of Eggs Broken by Earwigs	Mean Number of Intact Eggs
1:100	5.0 ± 1.41 ^e^	6.4 ± 1.51 ^c^	88.6 ± 1.52 ^a^
1:33	10.8 ± 1.48 ^d^	10.2 ± 1.48 ^b^	79.00 ± 1.87 ^b^
1:20	16.8 ± 2.77 ^c^	12.4 ± 0.55 ^b^	70.00 ± 2.95 ^c^
1:14	25.4 ± 2.97 ^b^	14.00 ± 2.65 ^a^	60.00 ± 3.65 ^d^
1:10	35.2 ± 3.96 ^a^	17.2 ± 4.60 ^a^	47.6 ± 4.72 ^e^
Mean	18.64 ± 11.18	12.04 ± 4.38	69.32 ± 14.82

Means within a column followed by different letters are significantly different (Duncan’s multiple range test: *p* ≤ 0.05).

**Table 2:** The number of larvae consumed by adult earwigs present in each ratio after 24 h under laboratory conditions.

Predator-Prey Ratios	Mean Number of Consumed Larvae by Earwigs	Mean Number of Dead Larvae	Mean Number of Remaining Live Larvae
1:100	2.8 ± 1.48 ^e^	11.4 ± 1.14 ^c^	85.4 ± 1.52 ^a^
1:33	5.4 ± 1.67 ^d^	13.8 ± 0.84 ^c^	80.8 ± 2.17 ^b^
1:20	9.0 ± 2.00 ^c^	34.0 ± 1.22 ^b^	57.4 ± 1.22 ^c^
1:14	18.0 ± 1.58 ^b^	35.2 ± 3.11 ^b^	46.8 ± 1.79 ^d^
1:10	26.4 ± 2.70 ^a^	38.4 ± 2.30 ^a^	35.2 ± 1.09 ^e^
Mean	12.32 ± 9.07	26.56 ± 11.88	61.04 ± 19.79

Means within a column followed by different letters are significantly different (Duncan’s multiple range test: *p* ≤ 0.05).

## Data Availability

All data sets of this work are available from corresponding author.
